# The relationship among vedolizumab drug concentrations, biomarkers of inflammation, and clinical outcomes in a Canadian real-world study

**DOI:** 10.1093/jcag/gwae010

**Published:** 2024-03-24

**Authors:** Cynthia H Seow, John K Marshall, Erin Stewart, Christopher Pettengell, Ryan Ward, Waqqas Afif

**Affiliations:** Inflammatory Bowel Disease Clinic, Division of Gastroenterology and Hepatology, Department of Medicine and Community Health Sciences, University of Calgary, Calgary, Alberta, Canada; Division of Gastroenterology, Department of Medicine and Farncombe Family Digestive Health Research Institute, McMaster University, Hamilton Ontario, Canada; Pentavere Research Group Inc., Toronto, ON, Canada; Pentavere Research Group Inc., Toronto, ON, Canada; Takeda Canada Inc., Toronto, ON, Canada; Division of Gastroenterology, McGill University Health Centre (MUHC), Montreal General Hospital, Montreal, QC, Canada

**Keywords:** inflammatory bowel disease, vedolizumab, therapeutic drug monitoring

## Abstract

**Background and Aims:**

Therapeutic drug monitoring is used to optimize anti-tumour necrosis factor biologic effectiveness in inflammatory bowel disease, but its role with other biological classes is unclear. This study explores relationships between post-induction vedolizumab trough concentrations and biochemical outcomes in a real-world study of individuals with inflammatory bowel disease.

**Methods:**

This retrospective analysis of data from a national patient support program between 2018 and 2020, included 436 individuals with Crohn’s disease or ulcerative colitis receiving vedolizumab. Optimal vedolizumab concentration thresholds (at weeks 6 and 14) were determined based on their ability to predict biochemical normalization (week 30 faecal calprotectin [<250 µg/g], C-reactive protein [<5 mg/l]). Thresholds best associated with each outcome were evaluated in multivariate analyses.

**Results:**

Among patients with Crohn’s disease, week 6 serum vedolizumab concentrations (>41.65 µg/ml) predicted normalization defined by C-reactive protein: Spearman correlation coefficient [*ρ*] = −0.26, *P* = 0.002 and multivariate analysis (MVA)—OR: 3.22, 95% CI: 1.32–7.87, *P* = 0.01, and at week 14 (>22.25 µg/ml): *ρ* = −0.38, *P* < 0.0001, and MVA—OR: 3.21, 95% CI: 1.26–8.17 but not faecal calprotectin. Similarly, among patients with ulcerative colitis, week 6 vedolizumab concentrations (>39.65 g/ml) predicted normalization defined by C-reactive protein: *ρ* = −0.26, *P* = 0.005 and MVA—OR: 4.03, 95% CI: 1.30–12.52, *P* = 0.016, and at week 14 (>17.35 µg/ml): *ρ* = −0.39, *P* = 0.0001 and MVA—OR: 6.95, 95% CI: 1.81–26.77, *P* = 0.005, but not faecal calprotectin.

**Conclusions:**

Induction and post-induction serum vedolizumab were not consistently associated with biochemical normalization. As such, proactive therapeutic drug monitoring for vedolizumab should not be routinely incorporated in a treat to target strategy for inflammatory bowel disease.

**Clinical Trial Registration Number:**

NCT04567628.

## Introduction

While anti-tumour necrosis factor (TNF) therapies have been a mainstay treatment of inflammatory bowel disease (IBD), newer biologic therapies directed against alternate molecular targets are now available for individuals with Crohn’s disease (CD) and ulcerative colitis (UC).^1–4^ With an increasing number of treatment options, there is a need to optimize use of each therapeutic class.^5^ Therapeutic drug monitoring (TDM) has been used to guide clinical decision-making after individuals experience a loss of response (ie, reactive TDM) or when aiming to predict treatment benefit or avoid loss of response (ie, proactive TDM). While both reactive and proactive TDM have been investigated as strategies to improve clinical outcomes in individuals receiving anti-TNF therapies, only reactive TDM is currently considered a standard of care.^6–10^ The value of TDM in other biologic treatment classes, however, is not well understood.^5,11–20^

Vedolizumab is a gut selective, anti-lymphocyte trafficking, monoclonal antibody against α_4_β_7_ integrin approved for the treatment of moderately to severely active UC and CD. Pivotal randomized, placebo-controlled trials in individuals with UC (GEMINI 1) and CD (GEMINI 2 and 3) demonstrated a relationship between increased serum vedolizumab trough concentrations (VTCs) and achievement of clinical remission in both induction and maintenance phases.^3,4,21–23^ Further post-hoc exploration of GEMINI 1 data found that individuals with UC with post-induction VTC levels in the upper quartile ranges had higher rates of deep remission at a year of follow-up, compared with those in the lowest quartile.^24,25^

Although subsequent smaller studies have evaluated proactive TDM, the role of routine monitoring of vedolizumab VTC in practice remains unclear.^12,13^ The optimal timing and target VTCs to predict clinical, endoscopic, or biochemical outcomes, and inform clinical management strategies, have yet to be established due to discrepant reports in the literature.^12,13,26–30^ Further studies leveraging larger and more diverse real-world populations are warranted. This analysis uses data captured by a Canada-wide patient support program to explore the value of post-induction VTC as a predictor of biochemical normalization, measured by faecal calprotectin (FCP) and C-reactive protein (CRP).

## Methods

### Study setting and population

All patients receiving commercial vedolizumab treatment in Canada participated in a nation-wide patient support program, developed by Takeda Canada Inc. to facilitate access and delivery of therapy. Sequential patients were included if they entered the patient support program between March 2018 and October 2020, during which time access to proactive TDM and biomarker testing from week 0 to week 30 was provided to all prescribing HCPs and patients receiving vedolizumab as part of a standard service offering.

### Cohort selection

Individuals received 300 mg of vedolizumab by intravenous infusion at weeks 0, 2, and 6 as induction. As maintenance therapy, individuals then received 300 mg of intravenous vedolizumab every 8 weeks. No patients in this cohort received subcutaneous vedolizumab. Treatment start date, end date (where relevant), and infusion interval were recorded. To maximize sample sizes for each analysis, individuals were grouped based on the availability of complete data for each predictor and outcome combination. The two predictors considered were weeks 6 VTC and 14 VTC. Outcomes explored for each predictor were week 30 FCP and week 30 CRP. This resulted in four analysis groups, each with complete data for a specific combination of predictor and outcome. Analysis group 1 included patients with complete week 6 VTC and week 30 CRP (CRP analysis group 1) or FCP (FCP analysis group 1). Analysis group 2 included patients with complete week 14 VTC and week 30 CRP (CRP analysis group 2) or FCP (FCP analysis group 2). Patients could have had complete data for multiple combinations of predictors and outcomes. In such cases, they were included in all relevant analysis groups.

### Clinical features

Baseline features collected included age, sex, disease type (CD or UC), duration of disease prior to starting vedolizumab treatment (years), prior biologic therapy (yes/no), CRP (mg/L), FCP (µg/g), albumin (g/l), and disease scores (Harvey–Bradshaw Index [HBI] for individuals with CD, and Partial Mayo Score [PMS] for those with UC). Blood measures were collected no more than one calendar day prior to vedolizumab infusion. Stool samples were within 2 weeks of infusion. If more than one sample was submitted within this window, a mean value was recorded. Serum VTC was measured using the vedolizumab Promonitor ELISA kit.

### Outcomes

Biochemical normalization at week 30 post vedolizumab treatment initiation was defined as CRP <5 mg/l or FCP <250 µg/g. Dose escalation was defined as a change of maintenance dose interval frequency from 8 weeks to 4 weeks.

### Statistical analysis

Descriptive statistics were used to characterize each analysis group. Continuous data were described using median and interquartile range (IQR) or mean and standard deviation depending on the distribution of the data; the Shapiro–Wilk test was used to test for normal distribution. Categorical variables were described using proportions in relation to their respective analysis group. No missing data were imputed.

### Outcome analyses

Relationships between VTC (weeks 6 or 14) and clinical outcomes were analysed using Spearman’s rank correlation coefficient. These relationships were further explored by comparing VTC (weeks 6 or 14) between individuals who did or did not achieve biochemical normalization, as measured by CRP or FCP using the Mann–Whitney *U* test. If a significant relationship was not found, no further analyses were completed. If a significant relationship was found, receiver-operating characteristic (ROC) curve analyses were constructed to establish the optimal cut-offs for vedolizumab TDM at week 6 and week 14 based on their prediction of CRP or FCP normalization. Univariate and multivariate analyses by logistic regression, using the determined thresholds, were performed to analyse potential factors that may influence the main outcome variables. Covariates were selected based on clinical significance and relationships described in existing literature.^29,31,32^ These included age (years), sex, week 0 albumin (g/l; only for TDM models), disease duration (years), and biologic treatment exposure.

### Sensitivity analyses

To understand the potential impact of including patients with FCP and CRP biomarker levels already below the defined thresholds at the time of first vedolizumab infusion, a sensitivity analysis was conducted in which patients were excluded from the Spearman’s correlation coefficient analyses if they had normalized biomarkers at baseline. Additionally, some patients received a week 10 dose of Vedolizumab. To account for this, an additional sensitivity analysis was conducted, excluding these patients from the Spearman’s correlation coefficient analyses.

### Ethical statement

This study was conducted in accordance with the requirements of the study protocol which was approved by Veritas Independent Review Board and also in accordance with: the ethical principles that have their origin in the Declaration of Helsinki; the International Conference on Harmonisation, E6 Good Clinical Practice: Consolidated Guideline; guidelines for good pharmacoepidemiology (GPP); and all applicable laws and regulations, including, without limitation, data privacy laws, clinical trial disclosure laws, and regulations, to protect the rights, safety, privacy, and well-being of study participants.

## Results

### Patient characteristics

A total of 436 individuals with IBD participated in the TDM offering of the patient support program between 2018 and 2020 and were included in the analyses (222 with UC and 214 with CD) (Table 1). Individuals with UC more often received vedolizumab as their first biological treatment than individuals with CD. At baseline, individuals with UC had higher levels of FCP than those with CD (Table 1).

**Table 1.** Baseline and clinicodemographics in all patients.

	Total study cohort (*N* = 436)	
	Individuals with Crohn’s disease (*N* = 214)	Individuals with ulcerative colitis (*N* = 222)
Sex (% female), *N* (%)	119 (55.6%)	102 (46.0%)
Age (years)		
*N*	214	222
Median (IQR)	48.5 (36.0, 61.0)	44.0 (32.0, 61.8)
Biologic treatment exposure (bio-naïve), *N* (%)	89 (41.6%)	120 (54.1%)
Disease duration (>2 years), *N* (%)	170 (79.4%)	159 (71.6%)
Week 0 albumin (g/l)		
*N*	178	150
Median (IQR)	42.0 (40.0, 44.0)	42.0 (40.0, 44.0)
Week 0 FCP (µg/g)		
*N*	92	91
Median (IQR)	522.0 (179.3, 1250.3)	977.0 (322.0, 3196.5)
Week 0 CRP (mg/l)		
*N*	185	155
Median (IQR)	3.0 (1.0, 7.0)	2.0 (1.0, 6.0)
Week 0 disease score	HBI	PMS
*N*	119	97
Median (IQR)	5.0 (3.0, 8.0)	4.0 (2.0, 6.0)

CRP, C-reactive protein; FCP, faecal calprotectin; HBI, Harvey–Bradshaw Index; PMS, Partial Mayo Score.

### Treatment outcomes

**Figure 1.**
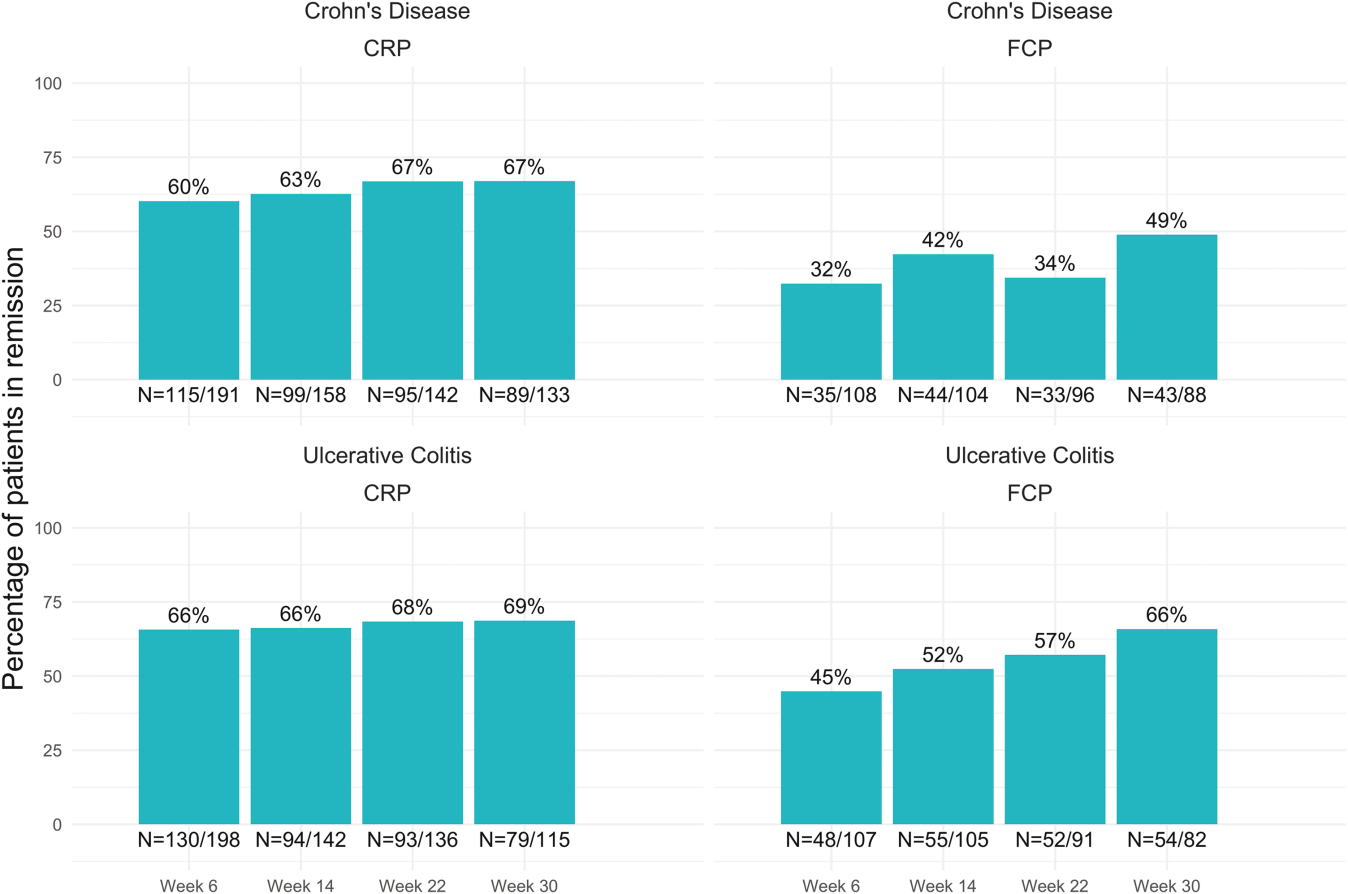
Percentage of patients in normalization by FCP and CRP. CRP, C-reactive protein; FCP, faecal calprotectin. Normalization defined as week 30 FCP <250 μg/g and CRP <5 mg/l.

The proportion of individuals with biochemical normalization as measured by CRP <5 mg/l rose in the early weeks of treatment and then remained stable, while the proportion with biochemical normalization as measured by FCP <250 µg/g increased progressively over the 30 weeks (Figure 1). 82% (358/436) of individuals with CD and UC in this study remained on vedolizumab treatment until at least week 30, and 28% (121/436) dose escalated prior to week 30. CRP was successfully tested and reported for 56% of included patients at week 30, and FCP was successfully tested and reported for 39% of included patients at week 30. Individuals with CD had higher measured Week 30 FCP levels than those with UC (Table 2).

**Table 2.** Biochemical and clinical outcomes and treatment information in all patients.

	Total study cohort (*N* = 436)	
	Individuals with Crohn’s disease (*N* = 214)	Individuals with ulcerative colitis (*N* = 222)
Week 30 FCP (µg/g)		
*N*	88	82
Median (IQR)	292.5 (86.3, 1005.0)	138.5 (41.5, 425.0)
Remitters *N* (%)	43 (48.9%)	54 (65.9%)
Week 30 CRP (mg/l)		
*N*	133	115
Median (IQR)	3.0 (1.0, 6.0)	2.0 (1.0, 5.0)
Remitters *N* (%)	89 (66.9%)	79 (68.7%)
Week 30 disease score	HBI	PMS
*N*	122	103
Median (IQR)	4.0 (2.0, 6.0)	1.0 (0.0, 3.0)
Remitters *N* (%)	74 (60.7%)	75 (72.8%)

CRP, C-reactive protein; FCP, faecal calprotectin; HBI, Harvey-Bradshaw Index; PMS, Partial Mayo Score.

Normalization defined as week 30 FCP <250 μg/g, CRP <5 mg/l, HBI <5, PMS <3.

### Week 6 VTC (analysis group 1)

There were 248 individuals (115 with UC and 133 with CD) with complete week 6 VTC and week 30 CRP measurements (“CRP analysis group 1”) and 170 individuals (82 with UC and 88 with CD) with complete data for week 6 VTC and week 30 FCP (“FCP analysis group 1”).

### CRP analysis group 1

Week 6 VTC was associated with week 30 CRP in individuals with CD (Spearman correlation coefficient [*ρ*] = −0.26, *P* = 0.002; Table S1). Individuals with CD achieving week 30 CRP normalization had higher week 6 VTC levels than those who did not (median [IQR]: 45.9 [31.8, 63.1] vs. 32.0 [26.8, 46.8], *P* =0.006; Table S2). The optimal week 6 VTC threshold best predicting CRP normalization at week 30 in individuals with CD was 41.65 µg/ml (AUROC [95% CI]: 0.65 [0.55, 0.75], sensitivity, specificity: 0.60, 0.66, *P* = 0.003; Table S3) which remained significant in multivariate analysis (OR: 3.22, 95% CI: 1.32–7.87, *P* = 0.010; Table 3).

**Table 3.** Multivariate analyses of week 6 VTC values using the determined threshold, potential covariates, and week 30 FCP or week 30 CRP.

	CRP analysis group 1				FCP analysis group 1	
	Individuals with Crohn’s disease		Individuals with ulcerative colitis		Individuals with Crohn’s disease	
Variables	OR (95% CI)	*P*-value	OR (95% CI)	*P*-value	OR (95% CI)	*P*-value
Week 6 VTC threshold FCP: >43.15 μg/ml (CD) CRP: > 41.65 μg/ml (CD) >39.65 μg/ml (UC)	3.22 (1.32, 7.87)	**0.010**	4.03 (1.30, 12.52)	**0.016**	1.59 (0.54, 4.72)	0.400
Age (years)	0.98 (0.96, 1.01)	0.268	1.002 (0.98, 1.03)	0.858	1.01 (0.97, 1.05)	0.662
Sex (female vs ***male***)	1.01 (0.44, 2.31)	0.983	0.69 (0.23, 2.02)	0.496	2.74 (0.94, 8.02)	0.065
Week 0 albumin (g/l)	0.97 (0.88, 1.06)	0.471	1.007 (0.92, 1.11)	0.880	1.24 (1.03, 1.49)	**0.026**
Disease duration (>2 years vs ≤2 ***years***)	1.17 (0.43, 3.13)	0.760	0.82 (0.27, 2.46)	0.726	0.84 (0.26, 2.76)	0.773
Biologic treatment exposure (bio-naïve vs *b****io-exposed***)	1.63 (0.69, 3.86)	0.266	1.15 (0.43, 3.09)	0.782	1.18 (0.40, 3.47)	0.761

CD, Crohn’s disease; CRP, C-reactive protein; FCP, faecal calprotectin; UC, ulcerative colitis; VTC, vedolizumab trough concentration.

Bold *P* values indicate statistical significance at *P* < 0.05.

Italicized variables are the reference for each analysis.

Among individuals with UC, week 6 VTC was associated with week 30 CRP (*ρ* = −0.26, *P* = 0.005; Table S1). Individuals with UC achieving normalization by week 30 CRP had higher week 6 VTC than those who did not (median [IQR]: 41.6 [28.7, 55.1] vs. 32.4 [16.8, 45.7], *P* = 0.019; Table S2). An optimal week 6 VTC threshold of 39.65 µg/ml predicted normalization by week 30 CRP in individuals with UC (AUROC [95% CI]: 0.64 [0.52, 0.75], sensitivity, specificity: 0.56, 0.72, *P* = 0.009; Table S3). This remained significant in multivariate analysis (OR: 4.03, 95% CI: 1.30–12.52, *P* = 0.016; Table 3).

### FCP analysis group 1

Week 6 VTC was associated with week 30 FCP among individuals with CD (*ρ* = −0.28, *P* = 0.0075; Table S1). Individuals with CD achieving normalization by week 30 FCP had higher week 6 VTC than those who did not (median [IQR]: 51.9 [37.7, 67.4] vs. 38.6 [26.9, 53.6], *P* = 0.007; Table S2). A week 6 VTC threshold of 43.15 µg/ml (AUROC [95% CI]: 0.67 [0.55, 0.78], sensitivity, specificity: 0.67, 0.67, *P* = 0.004) best predicted normalization by FCP in individuals with CD (Table S3), but this did not remain significant in multivariate analysis (Table 3). Baseline albumin was independently associated with week 30 FCP normalization in individuals with CD (OR: 1.24, 95% CI: 1.03–1.49, *P* = 0.026; Table 3).

Significant relationships between week 6 VTC and week 30 FCP were not observed in individuals with UC (Table S1).

### Week 14 VTC (analysis group 2)

There were 210 individuals (119 with CD and 91 with UC) with week 14 VTC and week 30 CRP (“CRP analysis group 2”) and 144 individuals (79 with CD and 65 with UC) with complete data for week 14 VTC and week 30 FCP (“FCP analysis group 2”).

### CRP analysis group 2

Week 14 VTC was significantly associated with week 30 CRP in individuals with CD (*ρ* = −0.38, *P* < 0.0001; Table S1). Individuals with CD who did achieve week 30 CRP normalization had higher week 14 VTC levels than those that did not achieve normalization (median [IQR]: 20.4 [12.7, 31.0] vs. 14.9 [8.5, 19.0] *P* = 0.004; Table S2). A week 14 VTC threshold of >22.25 µg/ml (AUROC [95% CI]: 0.66 [0.56, 0.76], sensitivity, specificity: 0.48, 0.83, *P* = 0.002) best predicted week 30 CRP normalization in individuals with CD (Table S3) and remained significant in multivariate analysis (OR: 3.21, 95% CI: 1.26–8.17, *P* = 0.010; Table 4).

**Table 4.** Multivariate analyses of week 14 VTC values using the determined threshold, potential covariates, and week 30 FCP or week 30 CRP.

	CRP analysis group 2				FCP analysis group 2	
	Individuals with Crohn’s disease		Individuals with ulcerative colitis		Individuals with Crohn’s disease	
Variables	OR (95% CI)	*P*-value	OR (95% CI)	*P*-value	OR (95% CI)	*P*-value
Week 14 VTC Threshold FCP: >18.10 μg/ml (CD) CRP: >22.25 μg/ml (CD) >17.35 μg/ml (UC)	3.21 (1.26, 8.17)	**0.010**	6.95 (1.81, 26.77)	**0.005**	2.19 (0.66, 7.32)	0.202
Age (years)	0.99 (0.96, 1.02)	0.350	0.99 (0.96, 1.02)	0.635	1.00 (0.96, 1.04)	0.840
Sex (female vs ***male***)	1.10 (0.46, 2.60)	0.843	0.88 (0.27, 2.84)	0.834	4.29 (1.29, 14.29)	**0.018**
Week 0 albumin (g/l)	1.00 (0.91, 1.09)	0.971	0.98 (0.88, 1.09)	0.710	1.26 (1.01, 1.57)	**0.040**
Disease duration (>2 years vs ≤2 ***years***)	1.63 (0.58, 4.63)	0.361	0.72 (0.20, 2.57)	0.611	1.14 (0.32, 4.00)	0.840
Biologic treatment exposure (bio-naïve vs ***bio-exposed***)	1.49 (0.60, 3.70)	0.401	1.13 (0.36, 3.57)	0.840	1.58 (0.47, 5.34)	0.462

CD, Crohn’s disease; CRP, C-reactive protein; FCP, faecal calprotectin; UC, ulcerative colitis; VTC, vedolizumab trough concentration.

Bold *P* values indicate statistical significance at *P* < 0.05.

Italicized variables are the reference for each analysis.

Among individuals with UC, week 14 VTC was significantly associated with week 30 CRP (*ρ* = −0.39, *P* = 0.0001; Table S1). Individuals with UC who achieved week 30 CRP normalization had higher week 14 VTC levels than those who did not (median [IQR]: 20.5 [10.8, 30.4] vs. 13.7 [8.4, 17.0], *P* = 0.006; Table S2). A week 14 VTC threshold of >17.35 µg/mL (AUROC [95% CI]: 0.68 [0.56, 0.80], sensitivity, specificity: 0.59, 0.82, *P* = 0.003) in individuals with UC best predicted week 30 CRP normalization (Table S3) and remained significant in multivariate analysis (OR: 6.95, 95% CI: 1.81–26.77, *P* = 0.005; Table 4).

### FCP analysis group 2

Week 14 VTC levels were significantly associated with week 30 FCP in individuals with CD (*ρ* = −0.32, *P* = 0.0036; Table S1). Individuals with CD achieving week 30 FCP normalization had higher measured week 14 VTC levels than those who did not (median [IQR]: 22.0 [14.8, 34.0] vs. 14.8 [9.4, 20.1], *P* = 0.004; Table S2). The determined optimal week 14 VTC threshold best predicting FCP normalization in individuals with CD was 18.10 µg/ml (AUROC [95% CI]: 0.69 [0.57, 0.81], sensitivity, specificity: 0.68, 0.71, *P* = 0.002; Table S3) but was not significant after controlling for clinically relevant covariates (Table 4). Baseline albumin (OR: 1.26, 95% CI:1.01–1.57, *P* = 0.040) and female sex (OR: 4.29, 95% CI: 1.29–14.29, *P* = 0.018), were independently associated with FCP normalization in individuals with CD (Table 4).

Among individuals with UC, no significant relationship was observed between week 14 VTC and week 30 FCP (Table S1).

When patients with normal FCP and CRP levels at the time of first vedolizumab infusion were excluded from analysis, Spearman correlation trends were similar to those estimated in the primary analysis (Table 5). Although not all *P*-values remained significant, this may have been due to a smaller sample size and resultant lack of power to demonstrate an effect.

**Table 5.** Sensitivity analysis of Spearman’s correlation between predictive variables in patients as per their week 30 FCP and week 30 CRP levels, excluding patients with FCP and CRP biomarker levels below the defined thresholds at time of first vedolizumab infusion.

	CRP analysis groups				FCP analysis groups			
	Individuals with Crohn’s disease		Individuals with ulcerative colitis		Individuals with Crohn’s disease		Individuals with ulcerative colitis	
	Spearman correlation coefficient	*P*-value	Spearman correlation coefficient	*P*-value	Spearman correlation coefficient	*P*-value	Spearman correlation coefficient	*P*-value
Week 6 VTC (analysis group 1)	−0.21	0.1	−0.12	0.39	−0.23	0.051	−0.21	0.076
Week 14 VTC (analysis group 2)	−0.37	**0.0062**	−0.43	**0.0067**	−0.31	**0.011**	0.0036	0.98

CRP, C-reactive protein; FCP, faecal calprotectin; VTC, vedolizumab trough concentration.

Units of measurement were as follows: VTC, μg/ml; FCP, μg/g; CRP, mg/l.

Table S4 shows results from the Spearman correlation conducted excluding patients who received a week 10 dose of Vedolizumab. Trends were consistent with those observed in the primary analyses and all *P*-values remained significant.

Additional analyses were conducted across all analysis groups, using an FCP normalization definition of <100 µg/g (data not shown) and similar results to those reported were observed: when comparing week 6 VTC analysis group 1, individuals with CD achieving week 30 FCP normalization also had higher week 6 VTC than those not with FCP normalization (*P* = 0.02 when using the FCP normalization definition of <100 µg/g, compared with *P* = 0.007 for FCP normalization definition of <250 µg/g).

## Discussion

This study explored the predictive value of clinical characteristics and proactive TDM in a large-scale real-world cohort of individuals with CD and UC treated with vedolizumab who participated in a Canada-wide patient support program. Optimal post-induction thresholds for VTC were determined based on their ability to predict week 30 biochemical normalization. VTC thresholds predicted week 30 CRP normalization, but not FCP normalization.

FCP and CRP are non-invasive biomarkers commonly used to guide clinical care in IBD. Serum CRP reflects systemic inflammation, with lower specificity and sensitivity for gastrointestinal inflammation. Conversely, FCP is a more direct measure of intestinal inflammation with high sensitivity, albeit with lower specificity for mucosal inflammation, but requires stool sampling and may have low adherence in practice.^33,34^ Both CRP and FCP are addressed in the STRIDE-II treatment guidelines, which recommend clinical response, biomarkers, endoscopic healing, and measures of quality of life as short, intermediate, and long-term therapeutic targets.^35^ The current study focused on biomarker outcomes, given their accessibility and patient acceptability, acknowledging that symptoms or patient-reported outcomes, while important, may be discordant with more objective markers of disease activity that are correlated with long-term disease outcomes. The analyses presented here suggest that pharmacokinetic predictors of normalization may differ between CD and UC and vary depending on the biochemical outcome of interest.

VTC at weeks 6 and 14 of treatment predicted week 30 CRP but not FCP normalization for both individuals with CD and UC. While the CRP cohorts analysed here were larger than the FCP cohorts, previously published data have also described an association between vedolizumab concentrations and either CRP alone or a composite measure of CRP and FCP.^36–40^ In this study, VTC levels at either time point did not correlate with week 30 FCP normalization in individuals with UC. Given that there were fewer individuals with FCP than with CRP data, this analysis may have been underpowered to demonstrate an association. Further, as per the STRIDE-II guidelines, normalization of CRP is considered a short-term treatment target, while a reduction in calprotectin to acceptable levels is deemed an intermediate treatment target. Therefore, outcome assessments at week 30 may not have allowed for adequate FCP decrement.^34^

Analyses from both GEMINI 1 and GEMINI 2 trials found that standard dosing of vedolizumab resulted in near-complete α_4_β_7_ receptor saturation.^3,4^ Subsequent studies have demonstrated that, in individuals with UC, vedolizumab concentrations in the colonic mucosa correlate with serum concentrations and suggest that non-response to vedolizumab is not necessarily due to inadequate tissue exposure and that dose escalation may not always be the solution to non-response.^41–43^

The ERELATE study reported that higher VTCs at weeks 6 and 10 were associated with clinical remission (defined as complete resolution of symptoms according to the local physician global assessment) at weeks 14 and 52, in UC and CD.^44^ CRP remission (using the same cut off of <5 mg/l we used in this present manuscript) at week 14 was associated with VTC ≥ 27.7 mg/l at week 6.^44^ The ENTERPRET study (NCT03029143) investigated the potential benefit of vedolizumab dose escalation in individuals with UC with high clearance of vedolizumab at week 5 and clinical non-response at week 6. Rates of clinical remission at week 30 were similar between individuals with dose escalation and individuals with standard dosing, suggesting a limited benefit of dose escalation in early non-responders with UC, as measured by endoscopic or clinical response.^45^ Similar results were observed in the TUMMY study, a large prospective observational study exploring the exposure-response relationship between VTC and clinical remission which observed no significant correlation between VTC and clinical remission across all patient groups, corroborating the potentially limited value of dose escalation based on VTC assessment.^30^

This study has several strengths and limitations. This study utilizes nationwide real-world data and encompasses all patients prescribed commercial vedolizumab in Canada from 2015 to 2020. This ensures that participants represent the diverse real-world IBD population, thereby augmenting the study’s generalizability. IBD patients in Canada have access to vedolizumab through both insurance and through a compassionate use program, further adding to the study’s generalizability. The large size of the cohort allowed for the comparison of multiple disease, predictor, and outcome combinations. Limitations associated with this study are typical of real-world datasets. This study used data that were collected to support access to vedolizumab treatment or voluntarily provided for research purposes. Notably, endoscopic data were not captured by the patient support program and therefore were not included in analyses. While all individuals analysed in this study came from the same patient support program population, they did differ slightly between analytic groups (while all patients could have had blood taken as a convenience sample at the infusion centre prior to therapy, there may be differences in individuals who did or did not submit faecal samples) thus caution is warranted when comparing results among analyses. Another potential limitation relates to the primary definition of biomarker normalization, which was defined irrespective of patients’ baseline FCP and CRP levels, meaning that some patients may have biomarker levels below the defined thresholds at time of first vedolizumab infusion. Even though the indication for vedolizumab is for the treatment of moderately to severely active CD and UC, some patients may not have exhibited an initial biochemical response if they were transferring from existing conventional therapies, immunomodulators of anti-TNF-alpha antagonists, due to intolerance or were already concurrent corticosteroids, in which the goal of vedolizumab would be to achieve corticosteroid free outcomes. To address this potential limitation, a sensitivity analysis was conducted, excluding patients with normal FCP and CRP at baseline (213 patients had normal CRP, 51 patients had normal FCP at baseline). The trends observed remained consistent with the primary analyses, suggesting limited impact on the study conclusions.

Additionally, it is possible that week 10 dosing of vedolizumab in a subset of patients may have impacted week 14 VTC. A sensitivity analysis was conducted excluding 30 individuals who received a week 10 dose. Spearman correlation coefficient analysis, trends, and *P*-values remained consistent, therefore not altering the conclusions of this study. Notably, endoscopic data, concomitant steroid exposure, prednisone use, or smoking exposure were not captured by the patient support program and therefore were not included in analyses.

Representative real-world data are required to understand vedolizumab TDM or other predictive tools in clinical practice. The results of this nationwide real-world study demonstrate that induction and post-induction serum vedolizumab concentrations are not consistently associated with biochemical (CRP and FCP) normalization in individuals with Crohn’s disease and ulcerative colitis. As such, this study does not support the use of proactive therapeutic drug monitoring, during induction, for vedolizumab as a treat to target strategy for those with IBD.

## Supplementary data

Supplementary data are available at *Journal of the Canadian Association of Gastroenterology* online.

gwae010_suppl_Supplementary_Materials

## Data Availability

The data underlying this article cannot be shared publicly due to the privacy of individuals that participated in the nation-wide patient support program, developed by Takeda Canada Inc. The data will be shared on reasonable request to the corresponding author.

## References

[1] Khan S , Rupniewska E, Neighbors M, Singer D, Chiarappa J, Obando C. Real-world evidence on adherence, persistence, switching and dose escalation with biologics in adult inflammatory bowel disease in the United States: a systematic review. J Clin Pharm Ther. 2019;44(4):495–507. 10.1111/jcpt.12830 30873648

[2] Singh S , Fumery M, Sandborn WJ, Murad MH. Systematic review with network meta-analysis: first- and second-line pharmacotherapy for moderate-severe ulcerative colitis. Aliment Pharmacol Ther. 2018;47(2):162–175. 10.1111/apt.14422 29205406

[3] Sandborn WJ , Feagan BG, Rutgeerts P, et al.; GEMINI 2 Study Group. Vedolizumab as induction and maintenance therapy for Crohn’s disease. N Engl J Med. 2013;369(8):711–721. 10.1056/NEJMoa1215739 23964933

[4] Feagan BG , Rutgeerts P, Sands BE, et al.; GEMINI 1 Study Group. Vedolizumab as induction and maintenance therapy for ulcerative colitis. N Engl J Med. 2013;369(8):699–710. 10.1056/NEJMoa1215734 23964932

[5] Papamichael K , Cheifetz AS. Therapeutic drug monitoring in inflammatory bowel disease: for every patient and every drug? Curr Opin Gastroenterol. 2019;35(4):302–310. 10.1097/MOG.0000000000000536 30973355 PMC6785387

[6] Papamichael K , Cheifetz AS. Therapeutic drug monitoring in IBD: the new standard-of-care for Anti-TNF therapy. Am J Gastroenterol. 2017;112(5):673–676. 10.1038/ajg.2017.21 28220781

[7] Mitrev N , Vande Casteele N, Seow CH, et al.; IBD Sydney Organisation and the Australian Inflammatory Bowel Diseases Consensus Working Group. Review article: consensus statements on therapeutic drug monitoring of anti-tumour necrosis factor therapy in inflammatory bowel diseases. Aliment Pharmacol Ther. 2017;46(11-12):1037–1053. 10.1111/apt.14368 29027257

[8] Cheifetz AS , Abreu MT, Afif W, et al. A comprehensive literature review and expert consensus statement on therapeutic drug monitoring of biologics in inflammatory bowel disease. Am J Gastroenterol. 2021;116(10):2014–2025. 10.14309/ajg.0000000000001396 34388143 PMC9674375

[9] Nguyen NH , Solitano V, Vuyyuru SK, et al. Proactive therapeutic drug monitoring versus conventional management for inflammatory bowel diseases: a systematic review and meta-analysis. Gastroenterology. 2022;163(4):937–949.e2. 10.1053/j.gastro.2022.06.052 35753383

[10] Sethi S , Dias S, Kumar A, Blackwell J, Brookes MJ, Segal JP. Meta-analysis: the efficacy of therapeutic drug monitoring of anti-TNF-therapy in inflammatory bowel disease. Aliment Pharmacol Ther. 2023;57(12):1362–1374. 10.1111/apt.17313 36495020

[11] Ma C , Battat R, Jairath V, Vande Casteele N. Advances in therapeutic drug monitoring for small-molecule and biologic therapies in inflammatory bowel disease. Curr Treat Options Gastroenterol. 2019;17(1):127–145. 10.1007/s11938-019-00222-9 30680599

[12] Yacoub W , Williet N, Pouillon L, et al. Early vedolizumab trough levels predict mucosal healing in inflammatory bowel disease: a multicentre prospective observational study. Aliment Pharmacol Ther. 2018;47(7):906–912. 10.1111/apt.14548 29384209

[13] Williet N , Boschetti G, Fovet M, et al. Association between low trough levels of vedolizumab during induction therapy for inflammatory bowel diseases and need for additional doses within 6 months. Clin Gastroenterol Hepatol. 2017;15(11):1750–1757.e3. 10.1016/j.cgh.2016.11.023 27890854

[14] Ward MG , Sparrow MP, Roblin X. Therapeutic drug monitoring of vedolizumab in inflammatory bowel disease: current data and future directions. Therap Adv Gastroenterol. 2018;11(1):1–10. 10.1177/1756284818772786 PMC5949937 29774052

[15] Al-Bawardy B , Ramos GP, Willrich MAV, et al. Vedolizumab drug level correlation with clinical remission, biomarker normalization, and mucosal healing in inflammatory bowel disease. Inflamm Bowel Dis. 2019;25(3):580–586. 10.1093/ibd/izy272 30165638

[16] Lee SD , Shivashankar R, Quirk D, et al. Therapeutic drug monitoring for current and investigational inflammatory bowel disease treatments. J Clin Gastroenterol. 2021;55(3):195–206. 10.1097/MCG.0000000000001396 32740098 PMC7960149

[17] Yarur AJ , Deepak P, Vande Casteele N, et al. Association between vedolizumab levels, anti-vedolizumab antibodies, and endoscopic healing index in a large population of patients with inflammatory bowel diseases. Dig Dis Sci. 2021;66(10):3563–3569. 10.1007/s10620-020-06669-6 33089483 PMC8449757

[18] Hanžel J , Sever N, Ferkolj I, et al. Early vedolizumab trough levels predict combined endoscopic and clinical remission in inflammatory bowel disease. United Eur Gastroenterol J. 2019;7(6):741–749. 10.1177/2050640619840211 PMC6620872 31316778

[19] Yarur AJ , Bruss A, Naik S, et al. Vedolizumab concentrations are associated with long-term endoscopic remission in patients with inflammatory bowel diseases. Dig Dis Sci. 2019;64(6):1651–1659. 10.1007/s10620-019-05570-1 30835029 PMC7057114

[20] Liefferinckx C , Minsart C, Cremer A, et al. Early vedolizumab trough levels at induction in inflammatory bowel disease patients with treatment failure during maintenance. Eur J Gastroenterol Hepatol. 2019;31(4):478–485. 10.1097/MEG.0000000000001356 30672828

[21] Sands BE , Feagan BG, Rutgeerts P, et al. Effects of vedolizumab induction therapy for patients with Crohn’s disease in whom tumor necrosis factor antagonist treatment failed. Gastroenterology. 2014;147(3):618–627.e3. 10.1053/j.gastro.2014.05.008 24859203

[22] Rosario M , French JL, Dirks NL, et al. Exposure-efficacy relationships for vedolizumab induction therapy in patients with ulcerative colitis or Crohn’s disease. J Crohns Colitis. 2017;11(8):921–929. 10.1093/ecco-jcc/jjx021 28333288

[23] Osterman MT , Rosario M, Lasch K, et al. Vedolizumab exposure levels and clinical outcomes in ulcerative colitis: determining the potential for dose optimisation. Aliment Pharmacol Ther. 2019;49(4):408–418. 10.1111/apt.15113 30663076 PMC6590294

[24] Singh S , Dulai PS, Vande Casteele N, et al. Systematic review with meta-analysis: association between vedolizumab trough concentration and clinical outcomes in patients with inflammatory bowel diseases. Aliment Pharmacol Ther. 2019;50(8):848–857. 10.1111/apt.15484 31483522 PMC7083298

[25] Guidi L , Pugliese D, Panici Tonucci T, et al. Early vedolizumab trough levels predict treatment persistence over the first year in inflammatory bowel disease. United Eur Gastroenterol J. 2019;7(9):1189–1197. 10.1177/2050640619873784 PMC6826518 31700632

[26] Vaughn BP , Yarur AJ, Graziano E, et al. Vedolizumab serum trough concentrations and response to dose escalation in inflammatory bowel disease. J Clin Med. 2020;9(10):3142. 10.3390/jcm9103142 32998473 PMC7601452

[27] Shmais M , Regueiro M, Hashash JG. Proactive versus reactive therapeutic drug monitoring: Why, When, and How? Inflammatory Intest Dis. 2022;7(1):50–58. 10.1159/000518755 PMC8820143 35224018

[28] Pouillon L , Rousseau H, Busby-Venner H, et al. Vedolizumab trough levels and histological healing during maintenance therapy in ulcerative colitis. J Crohns Colitis. 2019;13(8):970–975. 10.1093/ecco-jcc/jjz029 30698684

[29] Restellini S , Afif W. Update on TDM (Therapeutic Drug Monitoring) with ustekinumab, vedolizumab and tofacitinib in inflammatory bowel disease. J Clin Med. 2021;10(6):1242. 10.3390/jcm10061242 33802816 PMC8002563

[30] Sivridaş M , Creemers RH, Wong DR, et al. Therapeutic drug monitoring of vedolizumab in inflammatory bowel disease patients during maintenance treatment—TUMMY study. Pharmaceutics. 2023;15(3):972. 10.3390/pharmaceutics15030972 36986833 PMC10051381

[31] Carlsen A , Omdal R, Karlsen L, et al. Determination of lower cut-off levels of adalimumab associated with biochemical remission in Crohn’s disease. JGH Open. 2020;4(3):410–416. 10.1002/jgh3.12266 32514446 PMC7273736

[32] Papamichael K , Juncadella A, Wong D, et al. Proactive therapeutic drug monitoring of adalimumab is associated with better long-term outcomes compared with standard of care in patients with inflammatory bowel disease. J Crohns Colitis. 2019;13(8):976–981. 10.1093/ecco-jcc/jjz018 30689771 PMC6939875

[33] Khanna R , Wilson AS, Gregor JC, Prowse KL, Afif W. Clinical guidelines for the management of IBD. Gastroenterology. 2021;161(6):2059–2062. 10.1053/j.gastro.2021.09.021 34534537

[34] Pauwels RWM , Vries A.C. de, Woude C.J. van der. Faecal calprotectin is a reliable marker of endoscopic response to vedolizumab therapy: a simple algorithm for clinical practice. J Gastroenterol Hepatol. 2020;35(11):1893–1901. 32291796 10.1111/jgh.15063 PMC7687080

[35] Turner D , Ricciuto A, Lewis A, et al.; International Organization for the Study of IBD. STRIDE-II: an update on the selecting therapeutic targets in inflammatory bowel disease (STRIDE) Initiative of the International Organization for the Study of IBD (IOIBD): determining therapeutic goals for treat-to-target strategies in IBD. Gastroenterology. 2021;160(5):1570–1583. 10.1053/j.gastro.2020.12.031 33359090

[36] Dreesen E , Verstockt B, Bian S, et al. Evidence to support monitoring of vedolizumab trough concentrations in patients with inflammatory bowel diseases. Clin Gastroenterol Hepatol. 2018;16(12):1937–1946.e8. 10.1016/j.cgh.2018.04.040 29704680

[37] Ungaro RC , Yarur A, Jossen J, et al. Higher trough vedolizumab concentrations during maintenance therapy are associated with corticosteroid-free remission in inflammatory bowel disease. J Crohns Colitis. 2019;13(8):963–969. 10.1093/ecco-jcc/jjz041 31087100 PMC7185193

[38] Pouillon L , Vermeire S, Bossuyt P. Vedolizumab trough level monitoring in inflammatory bowel disease: a state-of-the-art overview. BMC Med. 2019;17(1):89. 10.1186/s12916-019-1323-8 31064370 PMC6505179

[39] Ungar B , Kopylov U, Yavzori M, et al. Association of vedolizumab level, anti-drug antibodies, and α4β7 occupancy with response in patients with inflammatory bowel diseases. Clin Gastroenterol Hepatol. 2018;16(5):697–705.e7. 10.1016/j.cgh.2017.11.050 29223444

[40] Plevris N , Jenkinson PW, Chuah CS, et al. Association of trough vedolizumab levels with clinical, biological and endoscopic outcomes during maintenance therapy in inflammatory bowel disease. Frontline Gastroenterol. 2020;11(2):117–123. 10.1136/flgastro-2019-101197 32133110 PMC7043080

[41] Van den Berghe N , Verstockt B, Gils A, et al. Tissue exposure does not explain non-response in ulcerative colitis patients with adequate serum vedolizumab concentrations. J Crohns Colitis. 2021;15(6):988–993. 10.1093/ecco-jcc/jjaa239 33245363

[42] Ungar B , Malickova K, Hanžel J, et al. Dose optimisation for loss of response to vedolizumab- pharmacokinetics and immune mechanisms. J Crohns Colitis. 2021;15(10):1707–1719. 10.1093/ecco-jcc/jjab067 33837762

[43] Pauwels RWM , Proietti E, van der Woude CJ, et al. Vedolizumab tissue concentration correlates to mucosal inflammation and objective treatment response in inflammatory bowel disease. Inflamm Bowel Dis. 2021;27(11):1813–1820. 10.1093/ibd/izab053 33705545 PMC8528144

[44] Vande Casteele N , Sandborn WJ, Feagan BG, et al. Real-world multicentre observational study including population pharmacokinetic modelling to evaluate the exposure-response relationship of vedolizumab in inflammatory bowel disease: ERELATE Study. Aliment Pharmacol Ther. 2022;56(3):463–476. 10.1111/apt.16937 35474325

[45] Yarur A , et al. A randomized trial of vedolizumab dose optimization in patients with moderate to severe ulcerative colitis who have early nonresponse and high drug clearance: the ENTERPRET trial [DDW abstract 791]. Gastroenterology. 2022;162(suppl 1):7–8. PMC9671057 36756650

