# Supplementary material for: The relationship among vedolizumab drug concentrations, biomarkers of inflammation, and clinical outcomes in a Canadian real-world study
Source: J Can Assoc Gastroenterol. 2024 Mar 24;7(4):290–8. doi: 10.1093/jcag/gwae010 (PMC11317629; doi:10.1093/jcag/gwae010)
Supplement: gwae010_suppl_Supplementary_Materials [file gwae010_suppl_supplementary_materials.zip › gwae010_suppl_Supplementary_Tables_1-4.docx]

**Supplementary Tables**

**Supplementary Table 1:** Spearman’s correlation between predictive variables in patients as per their Week 30 FCP and Week 30 CRP levels.

|  | **CRP Analysis Groups** | | | | **FCP Analysis Groups** | | | |
| --- | --- | --- | --- | --- | --- | --- | --- | --- |
|  | **Individuals with Crohn’s Disease** | | **Individuals with Ulcerative Colitis** | | **Individuals with Crohn’s Disease** | | **Individuals with Ulcerative Colitis** | |
|  | **Spearman correlation coefficient** | **p-value** | **Spearman correlation coefficient** | **p-value** | **Spearman correlation coefficient** | **p-value** | **Spearman correlation coefficient** | **p-value** |
| **Week 6 VTC (Analysis Group 1)** | -0.26 | **0.002** | -0.26 | **0.005** | -0.28 | **0.0075** | -0.21 | 0.055 |
| **Week 14 VTC (Analysis Group 2)** | -0.38 | **<0.0001** | -0.39 | **0.0001** | -0.32 | **0.0036** | -0.06 | 0.62 |

CRP, C-reactive protein; FCP, faecal calprotectin; VTC, vedolizumab trough concentration.

Units of measurement were as follows: VTC, μg/mL; FCP, μg/g; CRP, mg/L.

**Supplementary Table 2:** Comparison by Mann-Whitney U Test of predictive variables in patients as per their Week 30 FCP and Week 30 CRP levels.

|  | **CRP Analysis Groups** | | | | | | **FCP Analysis Groups** | | | | | |
| --- | --- | --- | --- | --- | --- | --- | --- | --- | --- | --- | --- | --- |
|  | **Individuals with Crohn’s Disease** | | | **Individuals with Ulcerative Colitis** | | | **Individuals with Crohn’s Disease** | | | **Individuals with Ulcerative Colitis** | | |
|  | **N** | **Median (IQR)** | **p-value** | **N** | **Median (IQR)** | **p-value** | **N** | **Median (IQR)** | **p-value** | **N** | **Median (IQR)** | **p-value** |
| Week 6 VTC (Analysis Group 1) | | | | | | | | | | | | |
| Week 30 FCP/CRP Remitters | 89 | 45.9 (31.8, 63.1) | **0.006** | 79 | 41.6 (28.7, 55.1) | **0.019** | 43 | 51.9 (37.7, 67.4) | **0.007** | 54 | 42.6 (29.9, 60.0 | 0.056 |
| Week 30 FCP/CRP Non-remitters | 44 | 32.0 (26.8, 46.8) |  | 36 | 32.4 (16.8, 45.7) |  | 45 | 38.6 (26.9, 53.6) |  | 28 | 33.2 (18.0, 51.7) |  |
| Week 14 VTC (Analysis Group 2) | | | | | | | | | | | | |
| Week 30 FCP/CRP Remitters | 79 | 20.4 (12.7, 31.0) | **0.004** | 63 | 20.5 (10.8, 30.4) | **0.006** | 37 | 22.0 (14.8, 34.0) | **0.004** | 41 | 20.8 (9.6, 30.9) | 0.886 |
| Week 30 FCP /CRP Non-remitters | 40 | 14.9 (8.5, 19.9) |  | 28 | 13.7 (8.4, 17.0) |  | 42 | 14.8 (9.4, 20.1) |  | 24 | 21.0 (10.2, 31.8) |  |

CRP, C-reactive protein; FCP, faecal calprotectin; VTC, vedolizumab trough concentration.
Normalization defined as Week 30 FCP <250μg/g or CRP <5mg/L.

Units of measurement were as follows: VTC, μg/mL; FCP, μg/g; CRP, mg/L.

**Supplementary Table 3:** AUROC analysis to determine optimal lthreshold of each predictive variable to predict normalization by Week 30 FCP and Week 30 CRP levels.

|  | **Week 30 CRP Analysis Groups** | | **Week 30 FCP Analysis Groups** |
| --- | --- | --- | --- |
|  | **Individuals with Crohn’s Disease** | **Individuals with Ulcerative Colitis** | **Individuals with Crohn’s Disease** |
| **Week 6 VTC (Analysis Group 1)** | | | |
| N | 133 | 115 | 88 |
| Predictive Threshold | 41.65 μg/mL | 39.65 μg/mL | 43.15 μg/mL. |
| AUROC (95% CI) | 0.65 (0.55, 0.75) | 0.64 (0.52, 0.75) | 0.67 (0.55, 0.78) |
| p-value | **0.003** | **0.009** | **0.004** |
| Sensitivity, specificity | 0.60, 0.66 | 0.56, 0.72 | 0.67, 0.67 |
| Number of patients above threshold | 68 | 54 | 44 |
| **Week 14 VTC (Analysis Group 2)** | | | |
| N | 119 | 91 | 79 |
| Predictive Threshold | 22.25 μg/mL | 17.35 μg/mL | 18.10 μg/mL |
| AUROC (95% CI) | 0.66 (0.56, 0.76) | 0.68 (0.56, 0.80) | 0.69 (0.57, 0.81) |
| p-value | **0.002** | **0.003** | **0.002** |
| Sensitivity, specificity | 0.48, 0.83 | 0.59, 0.82 | 0.68, 0.71 |
| Number of patients above threshold | 45 | 42 | 37 |

CRP, C-reactive protein; FCP, faecal calprotectin; VTC, vedolizumab trough concentration.
Normalization defined as Week 30 FCP <250μg/g or CRP <5mg/L.

|  | **CRP Analysis Groups** | | | | **FCP Analysis Groups** | | | |
| --- | --- | --- | --- | --- | --- | --- | --- | --- |
|  | **Individuals with Crohn’s Disease** | | **Individuals with Ulcerative Colitis** | | **Individuals with Crohn’s Disease** | | **Individuals with Ulcerative Colitis** | |
|  | **Spearman correlation coefficient** | **p-value** | **Spearman correlation coefficient** | **p-value** | **Spearman correlation coefficient** | **p-value** | **Spearman correlation coefficient** | **p-value** |
| **Week 6 VTC (Analysis Group 1)** | -0.24 | **0.0062** | -0.21 | **0.026** | -0.27 | **0.014** | -0.18 | 0.13 |
| **Week 14 VTC (Analysis Group 2)** | -0.38 | **<0.0001** | -0.40 | **0.00016** | -0.34 | **0.0026** | -0.089 | 0.5 |

**Supplementary Table 4:** Sensitivity analysis of spearman’s correlation between predictive variables in patients as per their Week 30 FCP and Week 30 CRP levels, excuding patients who received a Week 10 dose of Vedolizumab.

CRP, C-reactive protein; FCP, faecal calprotectin; VTC, vedolizumab trough concentration.

Units of measurement were as follows: VTC, μg/mL; FCP, μg/g; CRP, mg/L.
